# Predictors and Adverse Outcomes of Acute Kidney Injury in Hospitalized Renal Transplant Recipients

**DOI:** 10.3389/ti.2023.11141

**Published:** 2023-03-09

**Authors:** Tammy Hod, Bernice Oberman, Noa Scott, Liran Levy, Gadi Shlomai, Pazit Beckerman, Keren Cohen-Hagai, Eytan Mor, Ehud Grossman, Eyal Zimlichman, Moshe Shashar

**Affiliations:** ^1^ Renal Transplant Center, Sheba Medical Center, Ramat Gan, Israel; ^2^ Nephrology Department, Sheba Medical Center, Ramat Gan, Israel; ^3^ Sackler Faculty of Medicine, Tel-Aviv University, Tel Aviv, Israel; ^4^ Bio-Statistical and Bio-Mathematical Unit, The Gertner Institute of Epidemiology and Health Policy Research, Sheba Medical Center, Ramat Gan, Israel; ^5^ Institute of Pulmonary Medicine, Sheba Medical Center, Ramat Gan, Israel; ^6^ Department of Internal Medicine D and Hypertension Unit, The Division of Endocrinology, Diabetes and Metabolism, Sheba Medical Center, Ramat Gan, Israel; ^7^ Department of Nephrology and Hypertension, Meir Medical Center, Kfar Saba, Israel; ^8^ Central Management, Sheba Medical Center, Ramat Gan, Israel; ^9^ Department of Nephrology and Hypertension, Laniado Hospital, Netanya, Israel

**Keywords:** acute kidney injury, calcineurin inhibitors, readmission, renal transplant recipients, mortality abbreviations

## Abstract

Data about in-hospital AKI in RTRs is lacking. We conducted a retrospective study of 292 RTRs, with 807 hospital admissions, to reveal predictors and outcomes of AKI during admission. In-hospital AKI developed in 149 patients (51%). AKI in a previous admission was associated with a more than twofold increased risk of AKI in subsequent admissions (OR 2.13, *p <* 0.001). Other major significant predictors for in-hospital AKI included an infection as the major admission diagnosis (OR 2.93, *p* = 0.015), a medical history of hypertension (OR 1.91, *p* = 0.027), minimum systolic blood pressure (OR 0.98, *p* = 0.002), maximum tacrolimus trough level (OR 1.08, *p* = 0.005), hemoglobin level (OR 0.9, *p* = 0.016) and albumin level (OR 0.51, *p* = 0.025) during admission. Compared to admissions with no AKI, admissions with AKI were associated with longer length of stay (median time of 3.83 vs. 7.01 days, *p <* 0.001). In-hospital AKI was associated with higher rates of mortality during admission, almost doubled odds for rehospitalization within 90 days from discharge and increased the risk of overall mortality in multivariable mixed effect models. In-hospital AKI is common and is associated with poor short- and long-term outcomes. Strategies to prevent AKI during admission in RTRs should be implemented to reduce re-admission rates and improve patient survival.

## Graphical Abstract

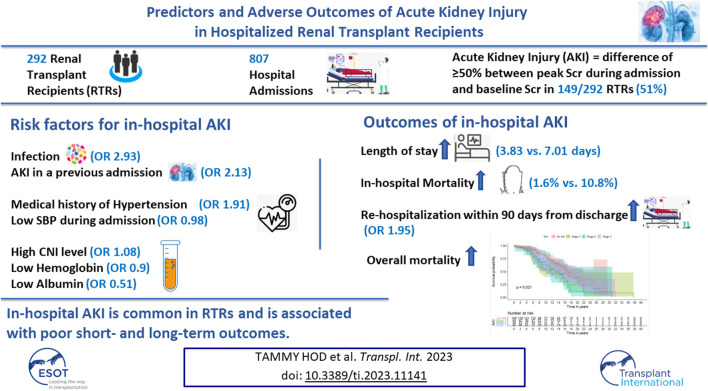

## Introduction

The prevalence of chronic kidney disease is increasing, accounting for more than 10% of hospital admissions in the adult population. The parallel increase in the rates of in-hospital acute kidney injury (AKI) may reach as much as 50% of intensive care unit (ICU) admissions (1, 2). The consequences of AKI during hospitalization are dismal (3, 4): Even modest changes in serum creatinine (Scr) (an increase >0.5 mg/dL) have been associated with a 6.5-fold increase in the odds of death and a 3.5-day increase in the length of stay (LOS) (5). Small changes in Scr have been also associated with increased mortality and prolonged hospitalizations in elderly patients admitted with congestive heart failure (6).

With the aim of preventing this serious complication, different studies have sought to establish predictors for AKI during hospitalization, both in the general population (7–11) and particularly for renal transplant recipients (RTRs). Unfortunately, however, information on predictors for AKI during hospitalization of RTRs is still lacking, although 11% of RTRs develop in-hospital AKI during the first three post-transplant years, which is associated with transplant failure and death (12). For RTRs, studies to date have focused mostly on delayed graft function, which is a form of AKI in the immediate peri-transplant period (13, 14).

RTRs constitute a unique population with an inherent increased risk vs. the general population for in-hospital AKI secondary to different etiologies related to subclinical and chronic rejection, higher risk of infections and immunosuppressive therapy. As a result, strategies to prevent or minimize the occurrence and consequences of AKI during hospitalization in this population would necessarily be more complex than those for the general population.

Renal allograft survival has improved significantly in the short term, with one-year graft survival rates reaching 98.4% (15). However, ensuring long-term graft survival still poses a very significant challenge in renal transplantation. For RTRs, a better understanding of the risk factors for AKI during admission would form the basis for developing preventive therapeutic measures aimed at reducing the rate of in-hospital AKI, resulting in improved long-term renal allograft survival.

In this study, we sought to pinpoint the risk factors for AKI during hospitalization of RTRs in a non-intensive care setting. In addition, we examined the implications of in-patient AKI for in-hospital mortality, duration of hospitalization, subsequent in-hospital AKI, re-hospitalizations, and overall mortality in this vulnerable population.

## Materials and Methods

### Study Population and Design

Clinical and biochemical parameters were collected retrospectively from the MdClone system, the data acquisition tool at Sheba Medical Center. Additional data was collected from clinical records, as needed. The study was approved by the local ethics committee (IRB approval number: SMC-70-5320). Data was collected for up to 12 admissions post “Renal Transplant” diagnosis for admission dates falling between July 2007 and November 2020.

**FIGURE 1:**
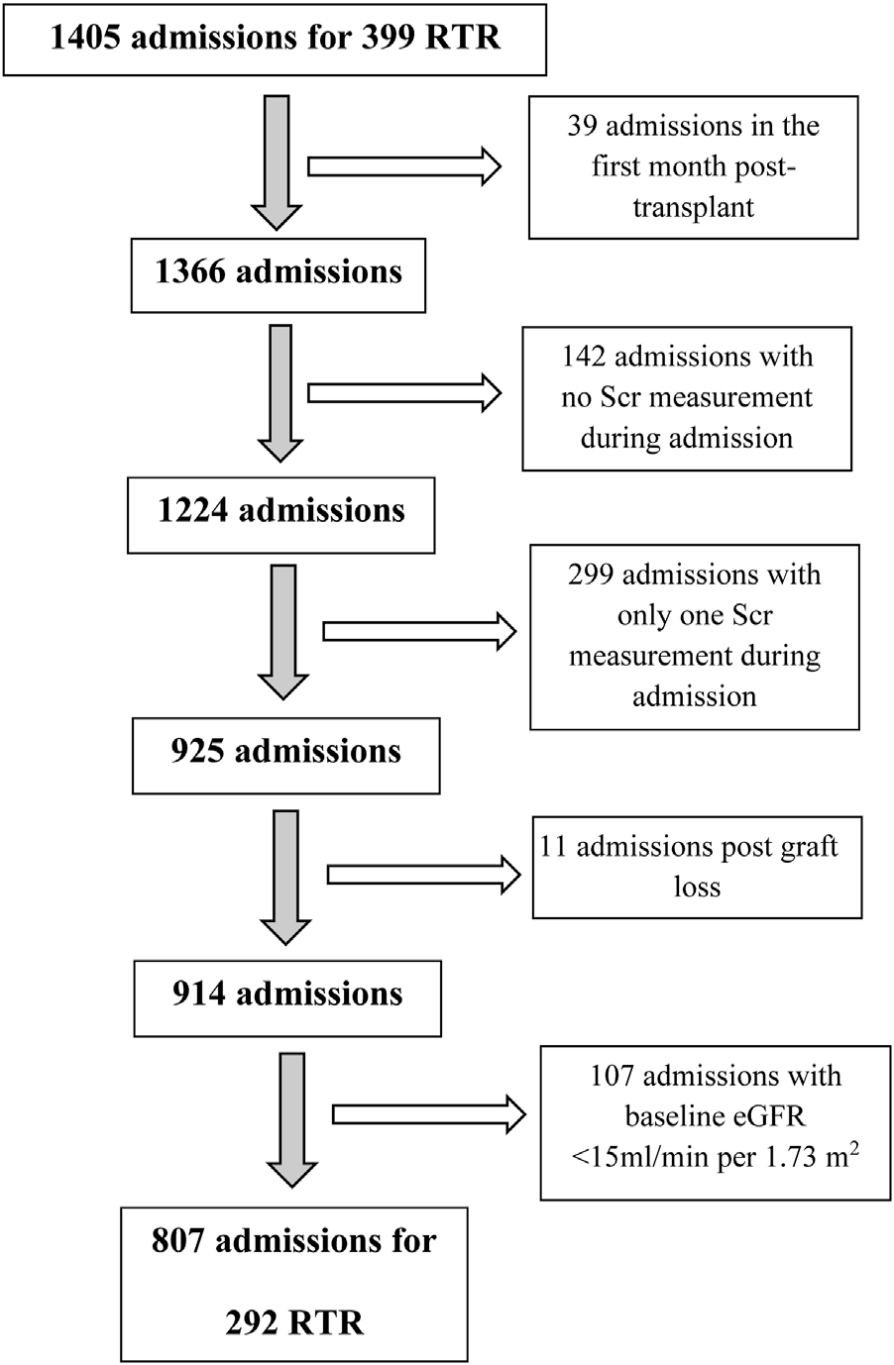
Consort diagram. RTR, renal transplant recipients; Scr, serum creatinine.

The initial dataset included 1405 hospitalizations for 399 transplant recipients. We then excluded from the analysis admissions post graft loss (baseline eGFR<15 mL/min per 1.73 m^2^), post chronic dialysis initiation, and/or admissions ≤30 days from transplant to eliminate the effect of changes in immunosuppressive medications or in renal allograft function early post-transplant secondary to slow and/or delayed graft function, infections and early rejections. Hospitalizations with no Scr or only one Scr measured during admission were also excluded (Figure 1). The final study cohort included 292 RTRs who had undergone kidney transplantation between June 1982 and June 2000, with a total of 807 non-ICU admissions.

### Primary Outcome

The primary outcome was AKI during admission, which was defined as a difference of ≥50% between peak Scr during admission and baseline Scr, according to the Kidney Disease Improving Global Outcomes (KDIGO) definition. AKI staging was based on the KDIGO definitions for SCr increases, i.e., a difference between peak Scr during admission and baseline Scr that was: Stage 1, ≥1.5–1.9 times the baseline Scr; Stage 2, ≥2–2.9 times the baseline Scr; and Stage 3, >3 times the baseline Scr or a peak Scr during admission ≥4.0 mg/dL or initiation of renal replacement therapy.

To determine baseline eGFR, we chose the minimum Scr in the 120 days to 1 day prior to admission. For admissions without Scr measurements in the 1- to 120-day period, we used minimum Scr during admission. To avoid misjudgment of baseline renal allograft function affected by frequent changes in Scr early post-transplant, we used minimum Scr during admission for baseline eGFR assessment in admissions of less than 150 days from transplant.

### Data Extraction and Study Assessments

The following information was extracted from electronic patient records: age, gender, etiology of end stage renal disease (ESRD), dialysis pre-transplant (yes/no), transplant number, donor type, transplant date and relevant medical history, specifically hypertension, congestive heart failure (CHF), ischemic heart disease (IHD) and pre-transplant diabetes. All diagnoses during admissions were obtained from MDClone. After a manual review of in-patient diagnoses, the main hospitalization etiology was selected, and diagnoses were grouped into five categories: infectious disease, cardiovascular disease, disease of the gastrointestinal system, neoplasm, and all others.

The following biochemical parameters during hospitalization were retrieved in an automated fashion from MDClone: average and maximum tacrolimus trough level, average total white blood cell count, average and minimum absolute lymphocyte count, average hemoglobin, average albumin, maximum and minimum glucose, maximum globulins, and average C-reactive protein. The following additional clinical and biochemical information during admission was also retrieved from MDClone: average and minimum systolic and diastolic blood pressures, average weight and body mass index (BMI), fever, maximum pulse, and minimum oxygen saturation. Use and average dose administered during admission for the following medications was automatically obtained from MDClone: tacrolimus, cyclosporine, mycophenolic acid (MPA) (for 238 admissions, mycophenolate dose was converted to the equivalent MPA dose by dividing mycophenolate dose by 1.388) and steroids (steroid derivatives used during admission, such as hydrocortisone, dexamethasone and methylprednisolone, were converted to the equivalent prednisone dose). Other medications administered during admission were also recorded.

### Statistical Analysis

All demographic, clinical and biochemical covariates of interest were tabulated and compared between patients for AKI during admission (yes/no) and between admissions (with and without AKI, and partitioned into AKI stage for AKI patients). Categorical variables were compared using the Chi-squared test (or Fisher’s test where the numbers were small), while continuous variables were first tested for normality using the Shapiro-Wilks Test (and for equality of variances), and were then compared using a t-test (or Anova) for normally distributed variables or a-parametric tests for non-normally distributed variables. An FDR [false discovery rate (Benjamini and Hochberg)] procedure was then carried out to correct for multiple comparisons.

For the primary outcome of AKI during admission, logistic mixed models were used, with RTR being a random effect, and other variables being fixed effects. Univariate models were considered first; variables that were significant (*p* = 0.05) and/or those with clinical importance were entered into multivariate models.

For the secondary outcomes, LOS was examined using a linear mixed model, with RTR as a random effect and the other variables being fixed effects. LOS (which is inherently right-tailed) was log-transformed to normalize it. Mortality during admission was modeled using the Cox proportional hazards model, which modeled the time from transplant to death or end of follow-up, taking into account the number of admissions per person. Overall mortality was modeled using Kaplan-Meier estimation, with AKI in different staging groups. Thereafter, the Cox proportional hazards model was used to model the time from transplant to death or end of follow-up. Readmission within 90 days was calculated. Logistic mixed models were used to predict the cause of readmission within 90 days, with RTR being a random effect, and other variables being mixed effects. For all secondary outcomes, univariate models were considered first. Significant variables and/or those with clinical importance were entered into multivariate models.

All statistical analyses were carried out using R-3.4.1 [R Core Team (2017). R: A language and environment for statistical computing. R Foundation for Statistical Computing, Vienna, Austria. URL https://www.R-project.org/].

## Results

### Characteristics of the RTRs Cohort

A total of 292 RTRs comprised our study cohort (Table 1). Median transplant age was 54.9 (IQR, 46.2–64.2); 98 (33.6%) were females. Median time from transplant to first admission was 6.33 years (IQR, 2.5–11.8). Of the RTR cohort, 64 (21.9%), 39 (13.4%), 59 (20.2%) and 26 (8.9%) patients had ESRD secondary to diabetic nephropathy, autosomal dominant polycystic kidney disease (APCKD), glomerulonephritis and nephrosclerosis, respectively; 158 (54.1%) were on renal replacement therapy before the transplant; and 140 (47.9%) had a living donor renal transplant. Forty RTRs (13.7%) died during admission, and the overall mortality rate for the duration of the study was 41.1%. The total number of admissions ranged between 1 and 10.

**TABLE 1:** Demographic and clinical characteristics of RTRs, stratified by AKI during admission.

Variable	Total cohort (*n* = 292)	Without AKI (*n* = 143)	With any AKI (*n* = 149)	*p*
RTR characteristics				
Transplant age, years [median (IQR)]	54.98 (46.2, 64.2)	55.53 (47.5, 64.51)	54.21 (45.2, 64.2)	0.89
Female sex, n (%)	98 (33.56)	48 (33.57)	50 (33.56)	1
Transplant to 1st admission, years [median (IQR)]	6.33 (2.45, 11.81)	6.7 (1.9, 11.2)	6.3 (3.2, 12.2)	0.35
ESRD etiology, *n* (%)				
ADPCKD	39 (13.36)	28 (19.58)	11 (7.38)	0.07
Diabetic nephropathy	64 (21.92)	31 (21.68)	33 (22.15)	
Glomerulonephritis	59 (20.21)	26 (18.18)	33 (22.15)	
Nephrosclerosis	26 (8.9)	12 (8.39)	14 (9.4)	
Other	69 (23.63)	29 (20.28)	40 (26.85)	
Unknown	35 (11.99)	17 (11.89)	18 (12.08)	
Pre-transplant dialysis				
Yes	158 (54.11)	72 (50.35)	86 (57.72)	0.11
No	46 (15.75)	29 (20.28)	17 (11.41)	
Unknown	88 (30.14)	42 (29.37)	46 (30.87)	
Transplant type, *n* (%)				
Kidney only	280 (95.89)	138 (96.5)	142 (95.3)	1
Liver kidney	4 (1.37)	2 (1.4)	2 (1.34)	
Heart kidney	7 (2.4)	3 (2.1)	4 (2.68)	
Pancreas kidney	1 (0.34)		1 (0.67)	
Transplant number, *n* (%)				
1	262 (89.73)	127 (88.81)	135 (90.6)	0.66
2	26 (8.9)	13 (9.09)	13 (8.72)	
3	4 (1.37)	3 (2.1)	1 (0.67)	
Donor type, *n* (%)				
Living	140 (47.9)	82 (57.34)	66 (44.3)	0.08
Deceased	85 (29.11)	36 (25.17)	49 (32.89)	
Unknown	59 (20.21)	25 (17.48)	34 (22.82)	
Number of admissions, *n* (%)				
1	116 (39.73)	86 (60.14)	30 (20.13)	<0.001**
2	55 (18.84)	29 (20.28)	26 (17.45)	
3	33 (11.3)	10 (6.99)	23 (15.44)	
4	31 (10.62)	9 (6.29)	22 (14.77)	
5	22 (7.53)	3 (2.1)	19 (12.75)	
6	15 (5.14)	3 (2.1)	12 (8.05)	
7	7 (2.4)	2 (1.4)	5 (3.36)	
8	9 (3.08)		9 (6.04)	
9	3 (1.03)	1 (0.7)	2 (1.34)	
10	1 (0.34)		1 (0.67)	
Number of AKI (per person), *n* (%)				
0		143 (100)		
1			76 (26.03)	
2			30 (10.27)	
3			25 (8.56)	
4			11 (3.77)	
5			2 (0.68)	
6			4 (1.37)	
8			1 (0.34)	
Death during admission, *n* (%)	40 (13.7)	5 (3.5)	35 (23.49)	<0.001**
Overall mortality, *n* (%)	120 (41.1)	40 (27.97)	80 (53.69)	<0.001**

*p < 0.05; **p < 0.01.

ADPKD, autosomal dominant polycystic kidney disease; ESRD, end stage renal disease, RTR, renal transplant recipient. Bold values are the *p* values which are significant (<0.05 or <0.01).

### Univariate Comparison of RTRs Without Any AKI vs. With Any AKI During Admission

Of the 292 RTRs, 149 (51%) had 1 to 8 AKI admission events. For patients with any AKI during admission, the number of admissions per person, the death rate during admission, and the overall mortality were all higher (*p* < 0.001). ESRD secondary to APCKD and renal living donor transplant were more common in RTRs without vs. with any AKI during admission as the difference between the groups approached statistical significance. All other comparisons of characteristics, including age, sex, time from transplant to first admission, transplant number and dialysis pretransplant, are shown in Table 1.

### Characteristics of Total Admissions

Our cohort of 292 RTR had a total of 807 non-ICU admissions. Median age during admission was 66.75 (IQR 57.16–73.12); 266 (33%) were females. Median time from transplant to admission was 7.65 years (IQR 4.22–12.75). The most prevalent admission etiology [312 (38.7%) admissions] was an infection. Forty (4.96%) admissions resulted in death during admission. Median LOS was 4.65 days (IQR 2.67–9). For 302 (37.4%) admissions, patients were readmitted within 90 days (Table 2).

**TABLE 2:** Demographic, clinical and biochemical characteristics of total admissions stratified by the presence or absence of AKI during admission.

Variable	Total admissions (*n* = 807)	Admissions without AKI (*n* = 510)	Admissions with AKI (*n* = 297)	*p* ^a^
RTR characteristics				
Admission age, years [median (IQR)]	66.75 [57.2, 73.1]	66.9 [58, 73.2]	66.5 [56, 73]	0.65
Female sex, *n* (%)	266 (33)	169 (33.1)	97 (32.7)	0.95
Transplant to admission, years [median (IQR)]	7.65 [4.2, 12.8]	7.6 [3.8, 12.6]	7.7 [4.8, 13.5]	0.43
ESRD etiology, *n* (%)				
ADPCKD	81 (10)	63 (12.4)	18 (6.1)	0.06
Diabetic nephropathy	187 (23.2)	119 (23.3)	68 (22.9)	
Glomerulonephritis	154 (19.1)	102 (20.0)	52 (17.5)	
Nephrosclerosis	73 (9)	40 (7.8)	33 (11.1)	
Other	212 (26.3)	124 (24.3)	88 (29.6)	
Unknown	100 (12.4)	62 (12.2)	38 (12.8)	
Pre-transplant dialysis				
Yes	449 (55.6)	289 (56.7)	160 (53.9)	0.75
No	125 (15.5)	79 (15.5)	46 (15.5)	
Unknown	233 (28.9)	142 (27.8)	91 (30.6)	
Transplant type, *n* (%)				
Kidney only	775 (96)	487 (95.5)	288 (97.0)	0.76
Liver kidney	7 (0.9)	5 (1.0)	2 (0.7)	
Heart kidney	23 (2.8)	17 (3.3)	6 (2.0)	
Pancreas kidney	2 (0.2)	1 (0.2)	1 (0.3)	
Transplant number, *n* (%)				
1	735 (91.1)	461 (90.4)	274 (92.3)	0.71
2	67 (8.3)	45 (8.8)	22 (7.4)	
3	5 (0.6)	4 (0.8)	1 (0.3)	
Donor type, *n* (%)				
Living	426 (52.8)	267 (52.4)	159 (53.5)	0.96
Deceased	239 (29.6)	152 (29.8)	87 (29.3)	
Unknown	142 (17.6)	91 (17.8)	51 (17.2)	
Medical history, *n* (%)				
Diabetes mellitus	273 (33.8)	169 (33.1)	104 (35.0)	0.7
Hypertension	496 (61.5)	297 (58.2)	199 (67.0)	0.037*
IHD	292 (36.2)	164 (32.2)	128 (43.1)	0.006**
CHF	170 (21.1)	98 (19.2)	72 (24.2)	0.19
Admission etiology, *n* (%)				
ID	312 (38.7)	170 (33.3)	142 (47.8)	<0.001**
CV	174 (21.6)	132 (25.9)	42 (14.1)	
GI	64 (7.9)	48 (9.4)	16 (5.4)	
CA	38 (4.7)	23 (4.5)	15 (5.1)	
Others	219 (27.1)	137 (26.9)	82 (27.6)	
Vital signs and other clinical parameters during admission, [median (IQR)]				
Fever max, °C	37.2 [36.9, 37.9]	37.2 [36.9, 37.6]	37.4 [37, 38.4]	<0.001**
Pulse max	96 [84, 110]	93 [81, 102]	103 [89, 120]	<0.001**
Pulse min	61 [54, 69]	61 [55, 70]	60 [53, 67]	0.04*
Pulse average	75.9 [68.1, 83.7]	75 [67, 82.5]	78 [70.1, 85.8]	<0.001**
SBP min mmHg	103 [89, 117]	108 [96, 120]	95 [80, 108]	<0.001**
SBP average mmHg	131.2 [118.8, 145.8]	132.6 [121.2, 147.1]	128.8 [115.5, 144.1]	0.008*
DBP min mmHg	54 [46, 62]	56 [50, 63]	50 [40, 59]	<0.001**
DBP average mmHg	70.7 [65.2, 76.6]	71 [66.4, 76.6]	69.8 [63.1, 76.6]	0.04*
O_2_ saturation min	93 [89, 95]	94 [90, 95]	91 [85, 94]	<0.001**
Weight average	75 [64.5, 87.4]	75 [65, 90]	73.8 [64, 85.1]	0.34
BMI average	26.4 [23.1, 30.5]	26.5 [23.4, 30.8]	26.1 [22.8, 30.3]	0.46
Medications during admission				
Tacrolimus, *n* (%)	386 (47.8)	232 (45.5)	154 (51.9)	0.17
Tacrolimus average dose mg [mean (SD)]	1.49 (0.96)	1.46 (0.92)	1.53 (1.03)	0.7
Cyclosporine, *n* (%)	97 (12.0)	63 (12.4)	34 (11.4)	0.79
Cyclosporine average dose, mg [mean (SD)]	63.51 (30.15)	60.60 (23.68)	68.90 (39.30)	0.3
MPA, *n* (%)	321 (39.8)	212 (41.6)	109 (36.7)	0.28
MPA average dose, mg [mean (SD)]	157.8 (217.4)	164.1 (218.7)	146.9 (215.1)	0.4
Steroids, *n* (%)	549 (68.0)	333 (65.3)	216 (72.7)	0.06
Steroids average dose, mg [mean (SD)]	16.01 (33.13)	12.27 (26.52)	22.10 (41.01)	<0.001**
mTOR inhibitors, *n* (%)	66 (8.2)	50 (9.8)	16 (5.4)	0.06
Azathioprine, *n* (%)	45 (5.6)	31 (6.1)	14 (4.7)	0.66
Loop diuretics, *n* (%)	339 (42)	193 (37.8)	146 (49.2)	0.005*
Thiazides, *n* (%)	44 (5.4)	29 (5.7)	15 (5.1)	0.79
Calcium channel blockers, *n* (%)	314 (38.9)	209 (41.0)	105 (35.4)	0.2
Beta blockers, *n* (%)	530 (65.7)	338 (66.3)	192 (64.6)	0.75
RAAS inhibitors, *n* (%)	247 (30.6)	176 (34.5)	71 (23.9)	0.005*
Aldosterone antagonists, *n* (%)	37 (4.6)	28 (5.5)	9 (3.0)	0.2
Statins, *n* (%)	387 (47.9)	249 (48.8)	138 (46.5)	0.69
NSAIDs, *n* (%)	5 (0.6)	2 (0.4)	3 (1.0)	0.48
PPIs, *n* (%)	550 (68.1)	327 (64.1)	223 (75.1)	0.004*
Laboratory results during admission [median (IQR)]				
White blood cell average (K/μL)	8.8 [6.5, 11.9]	8.3 [6.2, 10.8]	9.9 [7.3, 14.6]	<0.001**
Lymphocyte absolute average (K/μL)	1.1 [0.7, 1.6]	1.1 [0.8, 1.7]	1.1 [0.7, 1.5]	0.05
Lymphocyte absolute min (K/μL)	0.7 [0.4, 1.2]	0.8 [0.5, 1.3]	0.6 [0.3, 0.9]	<0.001**
Hemoglobin average (g/dL)	10.5 [9.3, 12]	11 [9.7, 12.3]	9.9 [9, 10.98]	<0.001**
Hemoglobin min (g/dL)	9.9 [8.3, 11.4]	10.5 [8.9, 11.9]	8.96 [7.66, 10.24]	<0.001**
Creatinine (mg/dL)	1.4 [1.0, 1.96]	1.3 [1.0, 1.8]	1.63 [1.13, 2.37]	<0.001
eGFR baseline (CKD-EPI)**	59.9 [41.3, 80.98]	61.2 [43.7, 81.1]	58.18 [35.81, 80.34]	0.34
Glucose max (mg/dL)	205 [137, 321.5]	188 [130, 282]	244 [152, 380]	<0.001**
Glucose min (mg/dL)	86 [71, 106]	90 [76, 112]	78 [64, 93]	<0.001**
Albumin average (g/dL)	3.2 [2.8, 3.6]	3.4 [3.0, 3.8]	2.9 [2.6, 3.3]	<0.001**
Albumin min (g/dL)	2.9 [2.5, 3.3]	3.1 [2.7, 3.5]	2.6 [2.3, 3]	<0.001**
Globulins max (g/dL)	2.9 [2.6, 3.3]	2.9 [2.5, 3.2]	3 [2.6, 3.6]	<0.001**
Globulins min (g/dL)	2.5 [2.1, 2.9]	2.5 [2.2, 2.9]	2.5 [2, 2.9]	0.47
Tacrolimus trough level average (μg/L)	5.5 [3.7, 8.2]	5.1 [3.6, 7.9]	6.0 [3.8, 8.6]	0.25
Tacrolimus trough level max (μg/L)	6.2 [4, 9.6]	5.7 [4.1, 8.5]	7.1 [4.1, 10.9]	0.028*
C-reactive protein average (mg/L)	61.3 [19.1, 113.3]	50.1 [14.8, 101.5]	79.8 [36.1, 137.8]	<0.001**
Death during admission, *n* (%)	40 (4.96)	8 (1.6)	32 (10.8)	<0.001**
LOS, days [median (IQR)]	4.6 [2.7, 9.0]	3.8 [2.1, 7.0]	7.01 [3.62, 15.34]	<0.001**
Readmission in 90 days, *n* (%)	302 (37.4)	148 (29.0)	154 (51.9)	<0.001**

^a^ After adjustment for multiple comparisons.

*p < 0.05; **p < 0.01.

***eGFR was calculated according to the following CKD-EPI formula: eGFR = 141* min (Scr/k, 1)α * max (Scr/k, 1)-1.209 * 0.993Age * 1.018 * 1.159 (if black) (where Scr - standardized serum creatinine; k = 0.7 if female, 0.9 if male; α = −0.329 if female, −0.411 if male; min = the minimum of Scr/k of 1; max = the maximum of Scr/k or 1).

ADPKD, autosomal dominant polycystic kidney disease; BMI, body mass index; CA, cancers; CHF, congestive heart failure; CV, cardiovascular disease; DBP, diastolic blood pressure; eGFR, estimated glomerular filtration rate; ESRD, end stage renal disease; GI, gastrointestinal; ID, infectious diseases; IHD, ischemic heart disease; LOS, length of stay; MPA, mycophenolic acid; NSAID, non-steroidal anti-inflammatory drug; RAAS, renin-angiotensin-aldosterone system; RTR, renal transplant recipients; SBP, systolic blood pressure. Bold values are the *p* values which are significant (<0.05 or <0.01).

### Univariate Comparison of Admissions Without vs. With AKI During Admission

An AKI during admission was recorded for 297 of 807 (36.8%) admissions. In admissions with AKI vs. admissions without AKI, ESRD secondary to APCKD was less prevalent, while nephrosclerosis was more common (*p* = 0.06). RTRs with at least one AKI had higher rates of hypertension and IHD (67% and 43.1% compared to 58.2% and 32.2% of admissions without AKI, *p* = 0.037 and 0.006, respectively). The main admission diagnosis was an infection in 142 (47.8%) of admissions with AKI vs. 170 (33.3%) in admissions without AKI (*p* < 0.001). Significant differences in the various vital signs monitored during admission [maximum temperature, maximum, minimum and average pulse, minimum and average systolic blood pressure (SBP) and diastolic blood pressure (DBP), and O_2_ saturation] were observed between admissions with and without AKI (Table 2). Steroids use and average dose during admission were significantly higher in admissions with vs. without AKI [216 (72.2%) vs. 333 (65.3%), *p* = 0.06 (after adjustment for multiple comparisons) and 22.1 (SD 41.01) vs. 12.27 (SD 26.52) mg, *p* < 0.001 respectively]. The use of loop diuretics and of proton pump inhibitors was also significantly higher in admissions with vs. without AKI, as opposed to the use of renin-angiotensin-aldosterone system (RAAS) inhibitors, which was lower in admissions with vs. without in-hospital AKI. Laboratory results also differed significantly between admissions with and without AKI. Total white blood cell count, maximum glucose, maximum globulins and C-reactive protein levels were higher in admissions with vs. without AKI, whereas lymphocytes (absolute minimum), average and minimum hemoglobin, minimum glucose and average and minimum albumin were lower in admissions with vs. without AKI. Maximum tacrolimus 12-h trough level was significantly higher in admissions with vs. without AKI [7.1 (IQR 4.05–10.9) vs. 5.7 (4.05–8.5), *p* = 0.028]. Rates of death during admission and readmission within 90 days as well as LOS were significantly higher for those with vs. without AKI (Table 2).

### Univariate Comparison of AKI Stages 1, 2 and 3 During Admission

Of 297 admissions with AKI during admission, 134 (45.1%), 70 (23.6%) and 93 (31.3%) presented with AKI Stages 1, 2 and 3, respectively. The rate of female RTRs fell as AKI progressed (39.6%, 31.4% and 23.7% in AKI stages 1, 2 and 3 respectively, *p* = 0.13). Time from transplant to admission increased from 6.95 (IQR 3.8–11.5) to 8.05 (IQR 4.65–12.3) to 8.7 years (IQR 5.9–16.5) as in-hospital AKI stage increased from 1 to 2 to 3, respectively (*p* = 0.04). Vital signs monitored during admission, such as maximum pulse, increased, whereas minimum SBP and minimum oxygen saturation decreased with worsening AKI stage. MPA use and average dose decreased, whereas average steroid dose increased with progression of AKI. Minimum glucose and average albumin decreased as in-hospital AKI progressed. LOS increased as AKI stage increased, but this was not statistically significant, possibly due to the low number of patients in each group. Rates of death during admission and readmission within 90 days increased as AKI worsened (Table 3).

**TABLE 3:** Demographic, clinical and biochemical characteristics of patients admitted with AKI, stratified by AKI stage during admission.

Variable	Admissions with AKI (*n* = 297)	AKI stage 1 (*n* = 134)	AKI stage 2 (*n* = 70)	AKI stage 3 (*n* = 93)	*p* ^a^
RTR characteristics					
Admission age, years, [median (IQR)]	66.5 [55.98, 73]	67.3 [58.6, 73.7]	66.1 [55.5, 75.5]	64.9 [53.4, 72]	0.4
Female sex, *n* (%)	97 (32.7)	53 (39.6)	22 (31.4)	22 (23.7)	0.13
Transplant to admission, years [median (IQR)]	7.7 [4.77, 13.48]	6.95 [3.8, 11.5]	8.05 [4.65, 12.30]	8.7 [5.9, 16.5]	0.04*
ESRD etiology, n (%)					
ADPCKD	18 (6.1)	10 (7.5)	5 (7.1)	3 (3.2)	0.29
Diabetic nephropathy	68 (22.9)	37 (27.6)	17 (24.3)	14 (15.1)	
Glomerulonephritis	52 (17.5)	25 (18.7)	9 (12.9)	18 (19.4)	
Nephrosclerosis	33 (11.1)	18 (13.4)	5 (7.1)	10 (10.8)	
Other	88 (29.6)	29 (21.6)	27 (38.6)	32 (34.4)	
Unknown	38 (12.8)	15 (11.2)	7 (10.0)	16 (17.2)	
Pre-transplant dialysis					
Yes	160 (53.9)	77 (57.5)	34 (48.6)	49 (52.7)	0.82
No	46 (15.5)	18 (13.4)	14 (20.0)	14 (15.1)	
Unknown	91 (30.6)	39 (29.1)	22 (31.4)	30 (32.3)	
Transplant type, *n* (%)					
Kidney only	288 (97.0)	130 (97.0)	68 (97.1)	90 (96.8)	0.86
Liver kidney	2 (0.7)	1 (0.7)	1 (1.4)	0 (0.0)	
Heart kidney	6 (2.0)	2 (1.5)	1 (1.4)	3 (3.2)	
Pancreas kidney	1 (0.3)	1 (0.7)	0 (0.0)	0 (0.0)	
Transplant number, *n* (%)					
1	274 (92.3)	124 (92.5)	67 (95.7)	83 (89.2)	0.6
2	22 (7.4)	9 (6.7)	3 (4.3)	10 (10.8)	
3	1 (0.3)	1 (0.7)	0 (0.0)	0 (0.0)	
Donor type, *n* (%)					
Living	159 (53.5)	72 (53.7)	36 (51.4)	51 (54.8)	0.55
Deceased	87 (29.3)	45 (33.6)	19 (27.1)	23 (24.7)	
Unknown	51 (17.2)	17 (12.7)	15 (21.4)	19 (20.4)	
Medical history, *n* (%)					
Diabetes mellitus	104 (35.0)	49 (36.6)	26 (37.1)	29 (31.2)	0.78
Hypertension	199 (67.0)	90 (67.2)	44 (62.9)	65 (69.9)	0.78
IHD	128 (43.1)	54 (40.3)	34 (48.6)	40 (43.0)	0.71
CHF	72 (24.2)	26 (19.4)	16 (22.9)	30 (32.3)	0.24
Admission etiology, *n* (%)					
ID	142 (47.8)	68 (50.7)	37 (52.9)	37 (39.8)	0.37
CV	42 (14.1)	22 (16.4)	5 (7.1)	15 (16.1)	
GI	16 (5.4)	7 (5.2)	6 (8.6)	3 (3.2)	
CA	15 (5.1)	5 (3.7)	2 (2.9)	8 (8.6)	
Other	82 (27.6)	32 (23.9)	20 (28.6)	30 (32.3)	
Vital signs and other clinical parameters during admission, [median (IQR)]					
Fever max, °C	37.4 [37, 38.40]	37.3 [37, 38.4]	37.4 [37, 38.25]	37.5 [37.1, 38.5]	0.49
Pulse max	103 [89, 120]	98 [87, 111]	103.5 [90.8, 128.5]	110 [94, 130]	0.01*
Pulse min	60 [53, 67]	60 [53.2, 67]	59.5 [53, 65]	61 [52, 68]	0.8
Pulse average	78 [70, 85.84]	76.6 [68, 84.6]	77.9 [71.5, 84.6]	79.2 [72.9, 87.1]	0.24
SBP min mmHg	95 [80, 108]	99 [86, 110.7]	91.5 [72, 102.8]	89 [70, 110]	0.01*
SBP average mmHg	128.8 [115.5, 144]	130 [118.7, 146.4]	128.4 [114.1, 137.5]	126.5 [112, 145.8]	0.04*
DBP min mmHg	50 [40, 59]8	50 [45, 58]	44 [39, 56.5]7	49 [36, 63]	0.24
DBP average mmHg	69.8 [63.1, 76.6]	70.5 [64.8, 75.3]	68.7 [62.6, 75.1]	70.6 [61.8, 81.1]	0.6
O_2_ saturation min	91 [85, 94]	93 [88, 95]	91 [84.3, 93]	90 [81, 94]	0.04*
Weight average, kg	73.8 [64, 85.1]	72.9 [63.2, 85]	72.7 [63.2, 91.2]	76.4 [65, 85.6]	0.78
BMI average	26.1 [22.8, 30.3]	26.1 [23.3, 29.5]	25.7 [22.1, 30.8]	26.6 [22.8, 30.5]	0.91
Medications during admission					
Tacrolimus, *n* (%)	154 (51.9)	68 (50.7)	47 (67.1)	39 (41.9)	0.03*
Tacrolimus average dose [mean (SD)]	1.53 (1.03)	1.49 (0.91)	1.50 (0.97)	1.62 (1.30)	0.88
Cyclosporine, *n* (%)	34 (11.4)	17 (12.4)	7 (10.0)	10 (10.8)	0.94
Cyclosporine average dose [mean (SD)]	68.90 (39.30)	71.81 (40.30)	64.03 (41.24)	67.35 (40.04)	0.92
MPA, *n* (%)	109 (36.7)	61 (45.5)	26 (37.1)	22 (23.7)	0.02*
MPA average dose [mean (SD)]	146.86 (215.11)	186.40 (237.78)	140.91 (192.53)	94.35 (185.05)	0.039*
Steroids, *n* (%)	216 (72.7)	92 (68.7)	53 (75.7)	71 (76.3)	0.59
Steroids average dose [mean (SD)]	22.10 (41.01)	14.47 (20.03)	24.98 (38.47)	30.72 (59.23)	0.06
mTOR inhibitors, *n* (%)	16 (5.4)	8 (6.0)	3 (4.3)	5 (5.4)	0.91
Azathioprine, *n* (%)	14 (4.7)	7 (5.2)	3 (4.3)	4 (4.3)	0.93
Loop diuretics, *n* (%)	146 (49.2)	61 (45.5)	34 (48.6)	51 (54.8)	0.59
Thiazides, *n* (%)	15 (5.1)	9 (6.7)	1 (1.4)	5 (5.4)	0.47
Calcium channel blockers, *n* (%)	105 (35.4)	41 (30.6)	29 (41.4)	35 (37.6)	0.47
Beta blockers, *n* (%)	192 (64.6)	83 (61.9)	43 (61.4)	66 (71.0)	0.53
RAAS inhibition, *n* (%)	71 (23.9)	35 (26.1)	15 (21.4)	21 (22.6)	0.82
Aldosterone antagonists, *n* (%)	9 (3.0)	4 (3.0)	1 (1.4)	4 (4.3)	0.72
Statins, *n* (%)	138 (46.5)	64 (47.8)	35 (50.0)	39 (41.9)	0.72
NSAIDs, *n* (%)	3 (1.0)	0 (0.0)	0 (0.0)	3 (3.2)	0.16
PPIs, *n* (%)	223 (75.1)	96 (71.6)	54 (77.1)	73 (78.5)	0.65
Laboratory results during admission [median (IQR)]					
White blood cell average (K/μL)	9.92 [7.27, 14.57]	9.5 [6.9, 14.1]	11.9 [7.7, 17.1]	10.2 [7.3, 13.5]	0.42
Lymphocyte absolute average (K/μL)	1.09 [0.67, 1.52]	1.1 [0.71, 1.5]	1.1 [0.7, 1.6]	1 [0.6, 1.35]	0.42
Lymphocyte absolute min (K/μL)	0.58 [0.28, 0.94]	0.6 [0.3, 1.1]	0.65 [0.3, 0.96]	0.5 [0.2, 0.8]	0.24
Hemoglobin average (g/dL)	9.91 [9.00, 10.98]	9.9 [8.98, 11]	10.1 [9.2, 11.1]	9.7 [8.8, 10.8]	0.59
Creatinine (mg/dL)	1.63 [1.13, 2.37]	1.4 [1.1, 1.9]	1.4 [1.1, 2]	2.5 [1.6, 3.5]	<0.001**
eGFR baseline (CKD-EPI)**	58.2 [35.8, 80.3]	59.6 [44, 77.8]	72.1 [54.5, 88.7]	34.3 [23.6, 72.6]	<0.001**
Glucose max (mg/dL)	244 [152, 380]	231 [152, 380]	268 [150, 405]	225 [168, 350]	0.78
Glucose min (mg/dL)	78 [64, 93]	81 [68, 103]	77 [65.25, 90]	74 [57, 85]	0.018*
Albumin average (g/dL)	2.9 [2.6, 3.3]	3.1 [2.7, 3.35]	2.8 [2.4, 3.1]	2.7 [2.4, 3.8]	0.003*
Globulins max (g/dL)	3 [2.6, 3.6]	3 [2.7, 3.6]	3.2 [2.8, 3.5]	3 [2.5, 3.6]	0.61
Tacrolimus trough level average (μg/L)	5.97 [3.8, 8.6]	6.5 [4.4, 9.1]	5.5 [3.5, 7.1]	4.95 [3.6, 8.1]	0.11
Tacrolimus trough level max (μg/L)	7.1 [4.1, 10.9]	8.1 [4.6, 11.2]7	6.2 [3.7, 8.5]	6.95 [3.9, 11.7]	0.39
C-reactive protein average (mg/L)	79.8 [36.1, 137.8]	62.7 [31.7, 119]	104.2 [42.9, 158.3]	85.5 [43.7, 140.5]	0.11
Death during admission, *n* (%)	32 (10.8)	3 (2.2)	9 (12.9)	20 (21.5)	<0.001**
LOS, days, [median (IQR)]	7 [3.6, 15.3]	6.31 [3.28, 13.11]	7.34 [4.3, 12.5]	8.17 [3.62, 19.95]	0.42
Readmission in 90 days, *n* (%)	154 (51.9)	65 (48.5)	31 (44.3)	58 (62.4)	0.042*

^a^ After adjustment for multiple comparisons.

*p < 0.05; **p < 0.01.

***eGFR was calculated according to the following CKD-EPI formula: eGFR = 141* min (Scr/k, 1)α * max (Scr/k, 1)-1.209 * 0.993Age * 1.018 * 1.159 (if black) (where Scr - standardized serum creatinine; k = 0.7 if female, 0.9 if male; α = −0.329 if female, −0.411 if male; min = the minimum of Scr/k of 1; max = the maximum of Scr/k or 1).

ADPKD, autosomal dominant polycystic kidney disease; BMI, body mass index; CA, cancers; CHF, congestive heart failure; CV, cardiovascular disease; DBP, diastolic blood pressure; eGFR, estimated glomerular filtration rate; ESRD, end stage renal disease; GI, gastrointestinal; ID, infectious diseases; IHD, ischemic heart disease; LOS, length of stay; MPA, mycophenolic acid; NSAID, non-steroidal anti-inflammatory drug; PPI, protein pump inhibitor; RAAS, renin-angiotensin-aldosterone system; RTR, renal transplant recipients; SBP, systolic blood pressure. Bold values are the *p* values which are significant (<0.05 or <0.01).

### Multivariable Analysis for AKI During Admission in RTRs

A mixed-effect logistic model (including admission age, sex, time from transplant to admission, ESRD etiology, medical history of diabetes, hypertension, IHD, CHF and AKI in a previous admission, admission etiology, vital signs, medications during admission, maximum tacrolimus trough level and other laboratory results during admission) revealed that the odds for an AKI during admission increased by 93% (OR 1.93, 95% CI 1.13–3.32, *p* = 0.017) for AKI in a previous admission and by 91% (OR 1.91, 95% CI 1.07–3.41, *p* = 0.028) for a medical history of hypertension. In addition, for every increase in minimum SBP of 1 mm Hg, the odds for in-hospital AKI decreased by 2% (OR 0.98, 95% CI 0.97–0.99, *p* = 0.002). Tacrolimus maximum trough level and albumin level during admission were also found to be associated with AKI during admission (OR 1.08, 95% CI 1.02–1.13, *p* = 0.005 and OR 0.51, 95% CI 0.29–0.92, *p* = 0.025, respectively). When tacrolimus maximum trough level was excluded to increase the number of patients and admissions included in the analysis, the odds for in-hospital AKI was more than twofold higher in the case of AKI in a previous admission (OR 2.13, 95% CI 1.44–3.14, *p* < 0.001) and almost three times higher when the major diagnosis upon admission was an infection (OR 2.93, 95% CI 1.23–6.98, *p* = 0.015). For every increase in minimum hemoglobin of 1 g/dL, the odds for AKI during admission decreased by 10% (OR 0.9, 95% CI 0.82–0.98, *p* = 0.016). Minimum SBP and albumin level during admission were also found to be independent predictors for in-hospital AKI (Table 4).

**TABLE 4:** Univariate and multivariate stepwise mixed effect logistic regression analysis for AKI during admission in RTRs.

Effect	Univariate logistic regression			Logistic regression (*n* = 193 patients, 385 admissions)			Logistic regression (*n* = 283 patients, 767 admissions)	
	Odds ratio (95% CI)	*p*		Odds ratio (95% CI)	*p*		Odds ratio (95% CI)	*p*
RTR characteristics								
Admission age, per 1 year increase	0.99 (0.98, 1.01)	0.85		0.99 (0.97, 1.02)	0.45		0.99 (0.97, 1.01)	0.21
Female vs. male	0.93 (0.59, 1.48)	0.77		1.17 (0.67, 2.04)	0.59		0.99 (0.65, 1.49)	0.95
Transplant to admission, years	1.02 (0.99, 1.06)	0.11		1.02 (0.97, 1.07)	0.47		1.00 (0.97, 1.03)	0.78
ESRD etiology								
APCKD	1			1			1	
Diabetic nephropathy	2.33 (1.00, 5.41)	0.049*		1.09 (0.34, 3.51)	0.88		1.06 (0.43, 2.57)	0.91
Glomerulonephritis	2.22 (0.94, 5.24)	0.07		1.7 (0.53, 5.51)	0.37		1.6 (0.7, 3.69)	0.27
Nephrosclerosis	3.41 (1.26, 9.22)	0.016*		1.87 (0.52, 6.73)	0.34		2.05 (0.77, 5.44)	0.15
Other	2.89 (1.26, 6.59)	0.012*		1.41 (0.48, 4.15)	0.53		1.72 (0.77, 3.82)	0.19
Unknown	2.36 (0.93, 6.00)	0.07		1.54 (0.43, 5.52)	0.51		1.7 (0.68, 4.23)	0.26
Medical history								
Diabetes mellitus	1.3 (0.82, 2.05)	0.27						
Hypertension	2.02 (1.27, 3.22)	0.003*		1.91 (1.07, 3.41)	0.028*		1.36 (0.88, 2.1)	0.16
IHD	1.95 (1.25, 3.05)	0.003*		1.23 (0.67, 2.23)	0.51		1.17 (0.75, 1.81)	0.49
CHF	1.59 (0.98, 2.57)	0.05		1.28 (0.64, 2.54)	0.49		1.35 (0.81, 2.24)	0.24
Previous AKI	2.88 (2.06, 4.03)	<0.001**		1.93 (1.13, 3.32)	0.017**		2.13 (1.44, 3.14)	<0.001**
Admission etiology								
CA	1			1			1	
CV	0.47 (0.19,1.14)	0.1		1.03 (0.26, 4.02)	0.97		1.5 (0.58, 3.9)	0.4
GI	0.51 (0.18, 1.42)	0.19		0.68 (0.16, 3.2.98)	0.61		0.82 (0.28, 2.39)	0.72
ID	1.34 (0.59, 3.05)	0.49		1.9 (0.55, 6.61)	0.31		2.93 (1.23, 6.98)	0.015*
Others	0.9 (0.39, 2.1)	0.81		2.2 (0.6, 8.14)	0.24		2.73 (1.11, 6.68)	0.03*
Vital signs and other clinical parameters during admission								
Pulse max, per 1/min increase	1.028 (1.02, 1.037)		<0.001**	1.00 (0.99–1.02)		0.64	1.01 (1.00, 1.02)	0.03*
SBP min, per 1 mm Hg increase	0.97 (0.96, 0.98)		<0.001**	0.98 (0.97, 0.99)		0.002*	0.98 (0.97, 0.99)	<0.001**
DBP min, per 1 mm Hg increase	0.95 (0.94, 0.97)		<0.001**					
Sat O_2_ min per 1% increase	0.97 (0.95, 0.99)		<0.001**	1.00 (0.98, 1.02)		0.95	1.01 (0.99, 1.02)	0.55
Medications during admission								
Steroids average dose, per 1 mg of prednisone increase	1.011 (1.005, 1.016)		<0.001**					
Loop diuretics use	1.52 (1.04, 2.22)		0.03*	1.22 (0.66, 2.26)		0.53	1.1 (0.71, 1.7)	0.66
PPI use	1.83 (1.2, 2.77)		0.0047**	1.05 (0.56, 1.94)		0.89	0.99 (0.64, 1.51)	0.95
Laboratory results during admission								
Lymphocyte absolute average per 1 K/μL increase	0.94 (0.82, 1.07)		0.36					
Lymphocyte absolute min per 1 K/μL increase	0.76 (0.6, 0.95)		0.015*	1.02 (0.87, 1.19)		0.79	1.0 (0.87, 1.15)	0.99
Hemoglobin average per 1g/dL increase	0.74 (0.67, 0.82)		<0.001**					
Hemoglobin min per 1g/dL increase	0.75 (0.69, 0.81)		<0.001**	0.95 (0.84, 1.08)		0.41	0.9 (0.82, 0.98)	**0.016**
eGFR baseline (CKD-EPI)** per 1 mL/min increase	0.999 (0.99, 1.01)		0.8	1.01 (0.99, 1.02)		0.44	1.00 (0.99, 1.01)	0.42
Glucose max per 1 mg/dL increase	1.005 (1.00, 1.01)		<0.001**	1.00 (1.00, 1.01)		**0.028**	1.00 (1.00, 1.01)	<0.001**
Glucose min per 1 mg/dL increase	0.98 (0.98, 0.99)		<0.001**					
Albumin average per 1 g/dL increase	0.59 (0.45, 0.8)		<0.001**					
Albumin min per 1 g/dL increase	0.2 (0.14, 0.28)		<0.001**	0.51 (0.29, 0.92)		**0.025**	0.42 (0.27, 0.64)	<0.001**
Globulins max per 1 g/dL increase	1.87 (1.36, 2.56)		<0.001**					
Globulins min per 1 g/dL increase	0.72 (0.5, 1.04)		**0.08**	1.02 (0.62, 1.69)		0.94	1.05 (0.72, 1.51)	0.81
Tacrolimus trough level max per 1 μg/L increase	1.065 (1.02, 1.11)		0.0065*	1.08 (1.02, 1.13)		0.005*		

*p < 0.05; **p < 0.01.

***eGFR was calculated according to the following CKD-EPI formula: eGFR = 141* min (Scr/k, 1)α * max (Scr/k, 1)-1.209 * 0.993Age * 1.018 * 1.159 (if black) (where Scr - standardized serum creatinine; k = 0.7 if female, 0.9 if male; α = −0.329 if female, −0.411 if male; min = the minimum of Scr/k of 1; max = the maximum of Scr/k or 1).

ADPKD, autosomal dominant polycystic kidney disease; CA, cancers; CHF, congestive heart failure; CV, cardiovascular disease; DBP, diastolic blood pressure; eGFR, estimated glomerular filtration rate; ESRD, end stage renal disease, GI, gastrointestinal; ID, infectious diseases; IHD, ischemic heart disease; PPI, protein pump inhibitor; RTR, renal transplant recipients; SBP, systolic blood pressure. Bold values are the *p* values which are significant (<0.05 or <0.01).

### Outcomes of AKI During Admission in RTRs

We examined four outcomes of AKI during admission: readmission within 90 days, mortality during admission, overall mortality, and LOS.

#### Readmission in 90 Days

In a mixed effect logistic regression analysis, in-hospital AKI increased the odds for readmission within 90 days by 95% (OR 1.95, 95% CI 1.35–2.81, *p* < 0.001). For every increase in minimum hemoglobin of 1 g/dL, the odds for readmission in 90 days decreased by 8% (OR 0.92, 95% CI 0.85–0.99, *p* = 0.02; Table 5).

**TABLE 5:** Multivariate mixed effect logistic regression analysis for readmission within 90 Days in RTRs.

Effect	Odds ratio (95% CI)	*p*
Admission age	1.01 (0.99–1.03)	0.17
Gender, F vs. M	0.93 (0.62–1.38)	0.72
Hypertension, yes vs. no	1.34 (0.91–1.97)	0.14
In-hospital AKI, yes vs.no	1.95 (1.35–2.81)	<0.001**
SBP min (for every increase of 1 mm Hg)	1.0 (0.99–1.01)	0.93
Albumin min per 1g/dL increase	0.76 (0.53–1.1)	0.15
Glucose max per 1 mg/dL increase	1.0 (0.99–1.0)	0.94
Hemoglobin min per 1 g/dL increase	0.92 (0.85–0.99)	0.02*

*p < 0.05; **p < 0.01.

RTR, renal transplant recipients; SBP, systolic blood pressure. Bold values are the *p* values which are significant (<0.05 or <0.01).

#### Mortality During Admission

In a mixed effect logistic regression analysis, admission age, AKI stage 3 vs. no AKI, minimum SBP and minimum albumin during admission were found to be independent predictors for in-hospital mortality. The odds of mortality during admission were four times higher in RTRs with AKI stage 3 vs. RTRs with no AKI during admission (OR 4.0, 95% CI 1.38–11.6, *p* = 0.01; Table 6).

**TABLE 6:** Multivariate mixed effect logistic regression analysis for mortality during admission in RTRs.

Effect	Odds ratio (95% CI)	*p*
Admission age (for every increase in 1 year)	1.05 (1.01–1.09)	0.01*
Gender, F vs. M	0.88 (0.35–2.18)	0.77
Reference- no AKI during admission		
AKI stage 1	0.65 (0.16–2.67)	0.55
AKI stage 2	2.76 (0.86–9.98)	0.09
AKI stage 3	4.00 (1.38–11.6)	0.01*
SBP min (for every increase of 1 mm Hg)	0.97 (0.95–0.98)	<0.001**
Albumin min per 1 g/dL increase	0.21 (0.08–0.55)	0.001**
Glucose max per 1 mg/dL increase	1.0 (0.99–1.0)	0.56
Hemoglobin min per 1 g/dL increase	0.99 (0.85–1.17)	0.94

*p < 0.05; **p < 0.01.

RTR, renal transplant recipients; SBP, systolic blood pressure. Bold values are the *p* values which are significant (<0.05 or <0.01).

#### Overall Mortality

**FIGURE 2:**
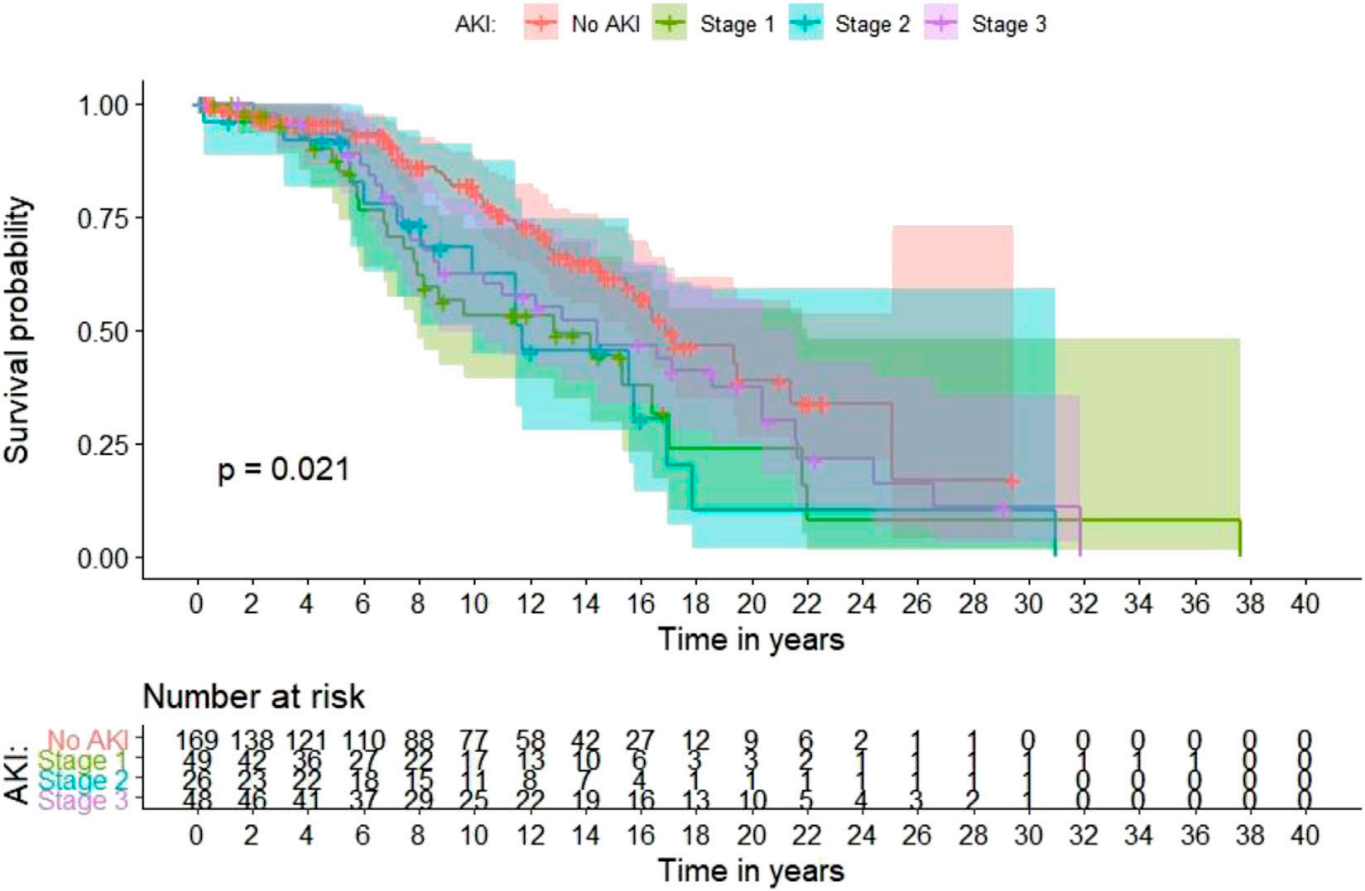
Long-term mortality based on the occurrence and severity of in-hospital AKI in the last admission for each patient.

Figure 2 shows Kaplan–Meier curves for time to death according to the presence and severity of AKI in the last admission for each patient. In a multivariable Cox proportional hazards regression model for long-term mortality, transplant age, diabetic nephropathy vs. all other ESRD etiologies, and presence of AKI vs. no AKI in any admission were associated with a 1.08-fold (95% CI 1.06–1.1), 1.95-fold (95% CI 1.27–2.99) and 1.51-fold (95% CI 1.01–2.25) increased risk of death, respectively (Table 7).

**TABLE 7:** Multivariate cox regression hazard model for overall mortality in RTRs.

Effect	Hazard ratio (95% CI)	*p*
Transplant age	1.08 (1.06–1.1)	<0.001**
Gender, F vs. M	0.93 (0.61–1.4)	0.72
Diabetic nephropathy vs. all other ESRD etiologies	1.95 (1.27–2.99)	0.002**
AKI, ever vs. never	1.51 (1.01–2.25)	0.04*

*p < 0.05;

**p < 0.01.

RTR, renal transplant recipients; ESRD, end stage renal disease. Bold values are the *p* values which are significant (<0.05 or <0.01).

#### Length of Stay

**FIGURE 3:**
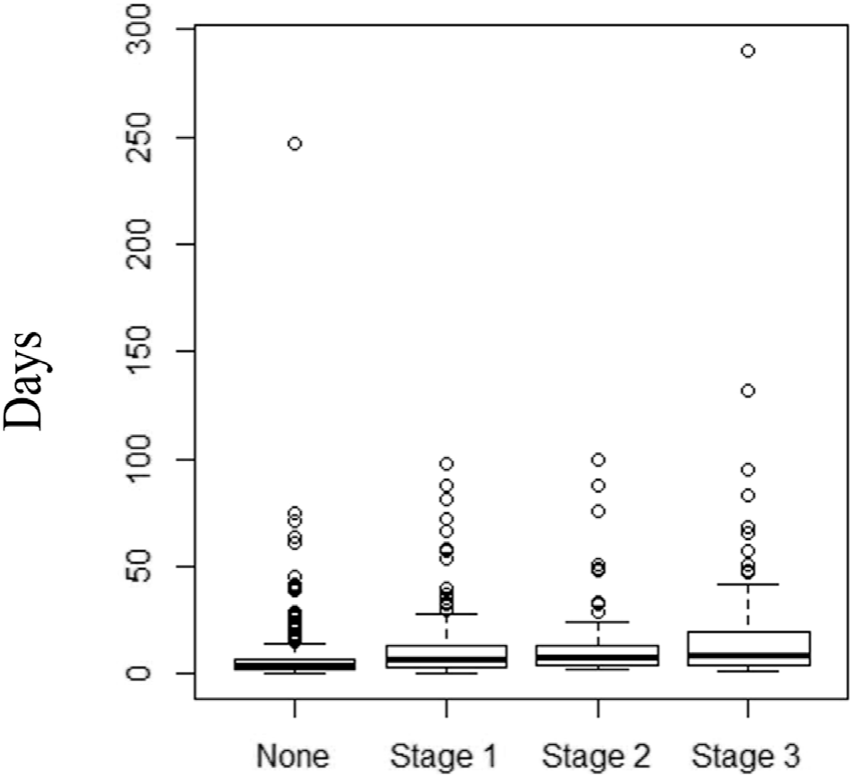
Box-plot diagram for in-hospital length of stay (days) in RTR with no AKI, and AKI stages 1,2 and 3.

Figure 3 shows a box-plot diagram for in-hospital LOS according to the presence and severity of in-hospital AKI. In a multivariable linear mixed model for LOS, a major admission diagnosis of a cancer significantly prolonged the LOS compared to all other admission etiologies. For every 1 mm Hg increase in minimum SBP, LOS was shortened by 1% (0.99, 95% CI 0.98–0.99, *p* < 0.001). Use of a calcineurin inhibitor (tacrolimus or cyclosporine) during admission increased the LOS by 41% (1.41, 95% CI 1.26–1.58, *p* < 0.001). Loop diuretic use, minimum hemoglobin, maximum glucose and minimum albumin during admission were independently associated with LOS. AKI during admission was not found to be an independent predictor for hospital LOS (Table 8).

**TABLE 8:** Multivariate linear mixed model for LOS during admission in RTRs.

Effect	Hazard ratio (95% CI)	*p*
Admission age (for every increase in 1 year)	0.99 (0.99–1.00)	0.003*
Gender, F vs. M	0.93 (0.81–1.06)	0.25
Diabetic nephropathy vs. all other ESRD etiologies	1.13 (0.96–1.33)	0.13
IHD, yes vs. no	1.1 (0.97–1.26)	0.13
AKI during admission yes vs. no	1.04 (0.92–1.18)	0.5
Major admission diagnosis		
CA	References	
CV	0.58 (0.44–0.76)	<0.001**
GI	0.57 (0.42–0.77)	<0.001**
ID	0.69 (0.53–0.89)	0.005*
SBP min (for every increase in 1 mm Hg)	0.99 (0.98–0.99)	<0.001**
Loop diuretics use	1.27 (1.12–1.43)	<0.001**
PPI use	1.05 (0.93–1.20)	0.4
CNI use	1.41 (1.26–1.58)	<0.001**
Hemoglobin min per 1 g/dL increase	0.97 (0.94–0.99)	0.01*
Glucose max per 1 mg/dL increase	1.00 (1.00–1.00)	**<0.001**
Albumin min per 1 g/dL increase	0.65 (0.57–0.73)	<0.001**

*p < 0.05; **p < 0.01.

CA, cancers; CNI, calcineurin inhibitors; CV, cardiovascular disease; GI, gastrointestinal disease; ID, infectious diseases; IHD, ischemic heart disease; PPI, protein pump inhibitor; RTR, renal transplant recipients; SBP, systolic blood pressure. Bold values are the *p* values which are significant (<0.05 or <0.01).

## Discussion

In this study of 292 RTRs, with a total of 807 non-ICU admissions, we found a 51% rate (149/292) of any AKI over multiple hospital admissions. AKI during admission was observed in 36.8% (297/807) of total admissions. Of 297 admissions with AKI, stages 1, 2 and 3 were recorded for 134 (45.1%), 70 (23.6%) and 93 (31.3%) admissions, respectively. Multivariable mixed effect models for AKI during admission revealed that an AKI in a previous admission doubled the odds for AKI in the subsequent admission. The odds for AKI during an admission were almost three times higher with major diagnosis of infectious etiology during admission. In addition, a medical history of hypertension, minimum SBP, minimum hemoglobin, albumin and tacrolimus maximum trough level were significantly associated with AKI during admission. AKI during admission was associated with adverse events, i.e., for patients who developed AKI, LOS and mortality during admission rose and rates of readmission within 90 days increased with worsening outcomes as AKI severity increased. The overall mortality risk was also higher in RTRs with any AKI vs. no AKI during admission.

The overall incidence of AKI developing 3 months or later after kidney transplantation, excluding RTRs with deceased donor transplants and recipients of second or third transplants, was 20.4% (16). In seeking to compare this finding with values in the literature, we found that there are only very few studies dealing with the subject. In pediatric kidney transplant recipients, the incidence of AKI was 37% over a study period of 12 years (17). A very much lower value – 3,066 of 27,232 transplant recipients (11.3%) – was reported in the only study focused on in-hospital AKI (4181 hospitalizations) during the first three post-transplant years. In that study, AKI was identified by the International Classification of Diseases, Ninth Revision, Clinical Modification (ICD-9-CM), which has limited sensitivity, and therefore the overall incidence of AKI was probably underestimated (12). Based on Scr levels pre and post admission, we detected AKI in a median time from transplant of 7.7 years (IQR 4.77–13.48) in 149/292 (51%) of RTRs, with a total of 513 admissions (1-10 admissions per person). Our analysis provides a more accurate assessment of the higher incidence of in-hospital AKI in RTRs compared to the non-transplant population, in which AKI occurs in 4%–20% of hospitalized patients (16, 18, 19). Similarly to our findings, a higher rate of AKI following cardiac surgery (46% vs. 28%) was observed in RTRs compared with non-RTRs (2).

In a study of 11,683 patients developing in-hospital AKI, 2954 (25%) were re-hospitalized with recurrent AKI within 12 months of discharge (20), with each episode of recurrence conferring an increased risk for progression to chronic kidney disease (21). Analysis of a large database of about 150,000 patients revealed that approximately 20% were readmitted with AKI and about 10% were seen in an emergency room within 30 days of discharge (22). We found the readmission rate within 90 days to be 51.9% vs. 29% in admissions without in-hospital AKI (*p* < 0.001). Moreover, the severity of AKI also affected the 90-day readmission rate, which reached 62.4% in stage 3 as opposed to 48.5% in stage 1 AKI. Our study is the first to show the negative effects of in-hospital AKI on the readmission rate and subsequent AKI events in RTRs.

It is not surprising that a major diagnosis of an infection was associated with in-hospital AKI; for example, in a study conducted in Italy the incidence of in-hospital AKI was 31.7% during the COVID-19 pandemic compared to 25.9% during the pre-COVID-19 period (23). RTRs are prone to infections and complications of infections, given the immunosuppressive agents they receive to prevent rejection. Infections are commonly complicated by AKI secondary to sepsis associated with hemodynamic instability, volume depletion, and the nephrotoxicity of antibiotics, among other factors. A medical history of hypertension, associated with oxidative stress and endothelial dysfunction (24), was also found to be an independent predictor for in-hospital AKI in our population, as previously described in patients with AKI following surgical resection of malignant pleural mesothelioma (25).

Given the large number and extensive variety of the components of our dataset (different vital signs, clinical parameters, medications and laboratory results during admission) that were retrieved as possible confounders, we were able to demonstrate associations of SBP, hemoglobin, albumin and maximum tacrolimus trough level with in-hospital AKI. CNI nephrotoxicity, a well-known complication of CNI use (26, 27), remains the leading cause of renal failure after transplantation of a non-renal organ (28, 29). Similar abnormalities have been found when CNIs are used in other settings, for example, in patients with psoriasis (30). The pathophysiology of acute CNI nephrotoxicity is related to profound alterations in renal vascular resistance and blood flow in the afferent and efferent arterioles and even a reduced diameter of the afferent arterioles (27). In line with our findings, higher vs. lower preoperative CNI trough levels (73% vs. 36%) have been associated with higher rates of AKI following cardiac surgery in RTRs (2).

The pathophysiology of low SBP and a low hemoglobin level associated AKI is related to the reduction in perfusion pressure and oxygenation, leading to ischemic injury. Renal ischemia-reperfusion injury in kidney transplantation leads to AKI, delayed graft function, and even graft loss (31). Kidney transplantation involves implantation of denervated kidneys, with impairment of blood flow autoregulation, rendering the renal allograft highly susceptible to ischemic injury and subsequent inflammation and cell death. A reduced nephron mass in RTRs may also increase their susceptibility to ischemic injury. Furthermore, renal allograft ischemia may exacerbate immune-related mechanisms of allograft injury, as manifested by the effect of cold and warm ischemia times on graft function and rejection (32).

AKI is common in RTRs and confers a high risk for graft failure and death (12, 17, 33). In populations other than RTRs, AKI has been associated with increased LOS and higher mortality (25, 34). We are the first to describe the association of in-hospital AKI in RTRs with increased LOS and mortality during admission. Our study is probably underpowered to detect an association between in-hospital mortality and milder AKI, found in studies of non-transplant patients (25). In addition, we found a strong association of AKI with overall mortality over a period of more than 30 years.

Several limitations should be mentioned, including the retrospective study design. In addition, minimum Scr during admission used as baseline Scr in recipients with no Scr within 120 days prior to admission or within 150 days from transplant may not reflect baseline Scr as it could be elevated due to AKI prior to admission, there is no information about the exact timing of maximum Scr during admission, rejection, use of erythropoietin stimulating agents, admissions to other hospitals, transplant loss, renal replacement therapy or recovery from AKI, mortality (death with a functioning graft) and death-censored graft loss. Urine output criteria were not used. In addition, the use of serum creatinine levels to estimate GFR has limitations in assessing kidney function. The strengths of this study include the large size of the cohort, creatinine-based definitions to capture index and recurrent AKI, and the power to examine multiple potential confounders. Nonetheless, we cannot exclude potential residual confounding as in-hospital AKI may be a surrogate for disease severity.

In conclusion, in-hospital AKI in RTRs is an independent risk factor associated with poor short- and long-term outcomes. RTRs with an AKI during admission should be followed up closely, with specific monitoring after discharge to reduce the risk of rehospitalization and death. Efforts should be made to identify patients at high risk for AKI, to develop strategies to prevent AKI during admission, and to minimize adverse outcomes. RTRs should be closely monitored during admission to prevent hypotension, anemia, and hypoalbuminemia. The association of CNI with in-hospital AKI further emphasizes the importance of individualized tailoring of immunosuppressive therapy based on rejection vs. infection risk to prevent complications associated with over immunosuppression, including infections, which are independently associated with in-hospital AKI and AKI itself.

## Data Availability

The raw data supporting the conclusion of this article will be made available by the authors, without undue reservation.

## Abbreviations

BMI, body mass index; CHF, congestive heart failure; CI, confidence interval; CNI, calcineurin inhibitor; DBP, diastolic blood pressure; DM, diabetes; eGFR, estimated glomerular filtration rate; ESRD, end stage renal disease; HTN, hypertension; ICU, intensive care unit; IHD, ischemic heart disease; KDIGO, Kidney Disease Improving Global Outcomes; LOS, length of stay; MPA, mycophenolic acid; OR, odds ratio; RTR, renal transplant recipients; SBP, systolic blood pressure; Scr, serum creatinine; SD, standard deviation.
